# Effect of theory-based education on promoting a healthy lifestyle in pre-diabetic women: RCT

**DOI:** 10.1186/s12905-022-01608-1

**Published:** 2022-02-04

**Authors:** Kolsoum MohammadniaMotlagh, Mohsen Shamsi, Nasrin Roozbahani, Mahmood Karimi, Rahmatollah Moradzadeh

**Affiliations:** 1 Student Research Committee, School of Health, Arak University of Medical Sciences, Arak, Iran; 2 Department of Health Education and Health Promotion, School of Health, Arak University of Medical Sciences, Arak, Iran; 3 Department of Public Health, School of Nursing and Midwifery, Saveh University of Medical Sciences, Arak, Iran; 4 Department of Epidemiology, School of Health, Arak University of Medical Sciences, Arak, Iran

**Keywords:** Pre-diabetes, Lifestyle, Planned behavior theory, Fasting blood sugar, Women

## Abstract

**Background:**

Due to the fact that pre-diabetic people are at higher risk of developing diabetes, it is possible to reduce the risk by taking preventive measures. Therefore, the present study aimed to determine the effect of theory-based education on promoting a healthy lifestyle and fasting blood sugar (FBS) in pre-diabetic women.

**Methods:**

This is a cluster‐randomized controlled trial that was performed on 71 pre-diabetic women referred to Arak Comprehensive Health Service Center. Thus, using cluster sampling method, one center was randomly assigned to the intervention group and one center to the control group. The data collection tool was a questionnaire based on the theory of planned behavior (TPB) and healthy lifestyle behavior that was completed before and at least 3 months after training and FBS test was performed. The experimental group received 3 training sessions of 60 to 90 min and finally the data were analyzed using statistical software.

**Results:**

After the intervention, the mean scores of knowledge (*P* < 0.001), attitude (*P* = 0.047) and perceived behavioral control related to physical activity (*P* = 0.046) and dietary function (*P* = 0.01) increased significantly in the intervention group. In addition, fasting blood sugar in the intervention group (99.70 ± 11.06) improved significantly compared to the control group (110.94 ± 17.09) (*P* = 0.003).

**Conclusion:**

Education based on the theory of planned behavior, by holding face-to-face meetings along with following up the samples after the educational intervention, can promote healthy lifestyle of pre-diabetic women. Therefore, designing and implementing similar interventions on all pre-diabetic individuals seem necessary.

*Trial registration*: The master's thesis in health education is approved by Arak University of Medical Sciences, Iran and is registered in the Iranian Registry of Clinical Trial (IRCT20190304042921N1). Prospectively registered 22/07/2019, https://en.irct.ir/trial/40596.

## Background

Pre-diabetes is defined as a condition in which a person's blood sugar level is higher than normal but not high enough to be diagnosed with diabetes [1]. The International Diabetes Federation reports that the worldwide prevalence of pre-diabetes is 471 million in 2035 [2].

Unchecked, the increasing prevalence of pre-diabetes can be predicted to only expand the numbers of people developing type 2 diabetes and all its associated health ramifications [3]. The International Diabetes Federation has reported the prevalence of diabetes mellitus worldwide, and by 2045, approximately 700 million people worldwide will have diabetes [4]. It is estimated that by 2030, around 9.2 million Iranians will develop diabetes [5].

Because the complications of diabetes are high in many countries; 27.2% of patients have macro vascular complications and 53.5% have micro vascular ones [6], prevention of which seems to be important. Based on research, the World Health Organization states that by changing lifestyle, up to 90% of type 2 diabetes can be prevented [7]. There is ample evidence that modest weight loss (5–7% of body weight) and increased engagement in physical activity can reduce or delay development of type 2 diabetes [3]. The fact that people with pre-diabetes are at risk for diabetes, although unavoidable, can be reduced by taking preventive measures [8]. Education is recognized as the most basic and important way to promote self-care behaviors as well as blood sugar control [9]. Given that human behavior is caused by various factors and health researchers in order to create a healthy lifestyle and design effective interventions to change behavior, need to know the behavior and the factors affecting it to change or modify existing behaviors and replace new behaviors. This determines the role of patterns and theories in the study of behavior [10], in this regard, one of the most widely used models for planning effective interventions is the theory of planned behavior (TPB), which is one of the patterns of behavior change [11].

Since the prevalence of metabolic syndrome and lack of exercise is higher in women and people living in urban areas [12] and also TPB can be a useful model for predicting physical activity behaviors and diet choices among women with pre-diabetes. The TPB is a well-known social cognitive theory in which intention is the immediate motivation of a certain behavior. It should be used as a lifestyle modifier to prevent the progression of diabetes in women with pre-diabetes [13], so we hypothesized that intervention with behavioral theories (TPB) is effective in improving the lifestyle of pre-diabetic women. The present study was conducted with the aim of the effect of theory-based education on promoting a healthy lifestyle and fasting blood sugar in pre-diabetic women.

## Methods

**Fig. 1:**
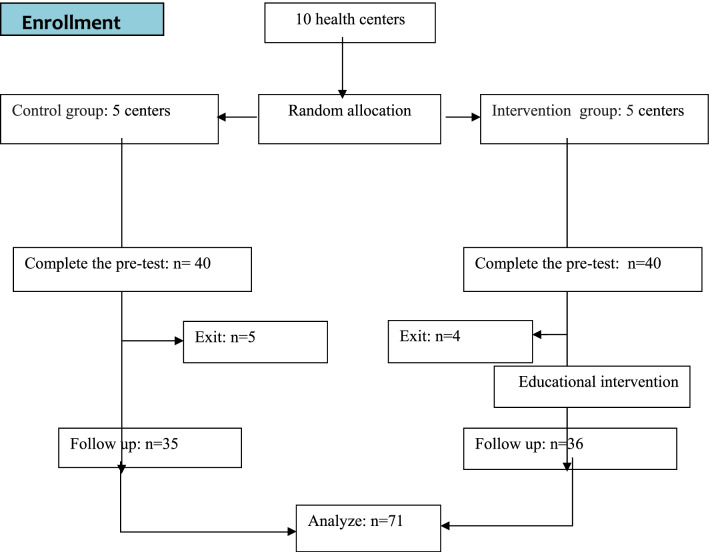
Diagram to demonstrate participant flow throughout the study. CONSORT flow diagram of the study design. This study will be conducted and reported on the basis of Consolidated Standards of Reporting Trials (CONSORT) 2010 statement. The figure shows the flow diagram of randomized controlled protocol

This study conducted and reported on the basis of Consolidated Standards of Reporting Trials (CONSORT) 2010 statement. A flow diagram of randomized controlled protocol is shown in Fig. 1.

Prospectively registered 22/07/2019, https://en.irct.ir/trial/40596. This study adheres to CONSORT guidelines.

The conceptual framework of conducting this study was that according to the study criteria, the samples were selected by referring to the health centers and randomly divided into control and intervention groups. Then pretest was administered to both groups and assessed FBS and the intervention group received training based on the TPB. Then the women’s were followed for three months and then posttest and FBS was administrated.

### Study design and sample size

This is a cluster‐randomized controlled trial with pre-test, post-test design (single blind) was performed on 71 pre-diabetic women referring to comprehensive health service centers in Arak, Iran, in 2020. For research, the city of Arak was divided into 5 classes based on socio-economic status and two health centers were selected from each section. Then, using the cluster sampling method, one center was randomly assigned to the intervention group and one center to the control group. At each center, samples were entered into the study by available methods. This study was single blind and participant who was blinded assignment to interventions and control groups. The study method, from data entry to data collection and follow-up, is presented in Fig. 1.

To determine the sample size based on study [14] and using the sample size formula and considering the means of 5 and 5.9, the standard deviation (SD) before and after the intervention were 1.66 and 0.9, respectively, based on the same study, α = 0.05, 0.2. = β and taking into account the 5% drop, the sample size of 80 people (intervention group: 40 people, control group: 40 people) was calculated.

Inclusion criteria included women 30 to 60 years old, fasting blood sugar equal to and more than 100 till equal to and less than 125, willingness to participate in research, ability to read and write (have at least 5 years of schooling), no pregnancy. Exclusion criteria were unwillingness to continue participating and becoming pregnant during the study.

In this study, primary outcomes included promoting a healthy life style and secondary outcome included changes in knowledge, attitude, subjective norms, perceived behavioral control, behavioral intention, in pre-diabetic women’s.

### Measurement

To collect information, a questionnaire including demographic variables, knowledge, sports behavior and diet, TPB constructs on physical activity and diet were used before and at least 3 months after the intervention. FBS was also measured before and after the intervention in a single laboratory. The knowledge section with content validity ratio (CVR) and content validity index (CVI)indices was confirmed by expert professors and Cronbach's alpha coefficient of 0.7, which included 10 multiple choice questions. The total score was calculated from zero to one. The validity and reliability of the structures of the theory of planned behavior for a healthy lifestyle including physical activity [13] and diet (fruit and vegetable consumption, and the intake of foods low in saturated fat) [13, 15] have also been confirmed in previous studies. Attitude questions (6 questions per behavior), Subjective norms (3 questions per behavior), Perceived behavioral control (4 questions per behavior), Intention (1 question per behavior) were scored from 1 point (strongly disagree)to 5 points (strongly agree)using the 5-point Likert scale. This questionnaire was validated by Rahmati-Najarkolaei et al. to Persian language [13].

The following section provide some of TPB questionnaire for physical activity and healthy eating.

## TPB questionnaire for physical activity

### Attitude

The attitude toward PA was measured questions on a 5-point Likert scale ranging from 1 (strongly disagree) to 5 (strongly agree). For example: “for me to exercise for at least 30 min, 5 days per week at a moderate intensity over the next months would be …”.

### Subjective norm

Subjective norm was determined using three items on a 5-point Likert scale ranging from 1 (strongly disagree) to 5 (strongly agree). For example, ‘People who are important to me would approve of me exercising for at least 30 min, 5 days per week at a moderate intensity over the next month.”

### Perceived behavioral control

For example: “Exercising for at least 30 min, 5 days per week at a moderate intensity over the next month would be…”.

### Behavioral intention

For example, ‘I intend to exercise for at least 30 min, 5 days per week at a moderate intensity over the next months’.

## TPB questionnaire on healthy eating

### Attitude

The participants were asked to indicate their attitude towards eating food low in saturated fat or sugar. For example “For me eating healthy foods daily over the next months would be …’.

### Subjective norms

Fore example “ people who are important to me would approve of me eating healthy food daily over the next months’.

### Perceived behavioral control

The participants control/confidence over eating healthy food daily was assessed using Likert-type items. For example, ‘I have complete control over whether I eat healthy food daily during the next months’. All responses were scored by a 5-point Likert scale ranging from 1 (strongly disagree) to 5 (strongly agree).

### Behavioral intention

The intention to eat healthy food was measured by questions regarding one’s willingness to eat healthy food over the next months. For example, ‘In the next months, I intend to eat healthy food everyday’. The responses were scored by a 5-point Likert scale ranging from 1 (strongly disagree) to 5 (strongly agree).

Physical activity was measured using a valid version of the Godin Leisure Exercise Questionnaire (GLTEQ) [16]. This questionnaire was validated by Rahmati-Najarkolaei et al. to Persian language [13]. In this questionnaire, participants were asked to show the average duration (in minutes) and frequency of mild, moderate and intense physical activity per week during the past month. Participants' responses were converted to metabolic equivalent of task (MET) scores (in MET-Time-week), which is the equivalent of metabolic energy during physical activity, using the following formula: ∑ [(Mild × 3) + (Moderate × 5) + (Severe × 9)].

MET-Time less than 14 indicates inactivity or inadequate activity (zero score), between 14 and 23 indicates moderate activity (score one), 24 and above indicates adequate activity (Score two) and the total score was calculated between zero and two. Dietary behavior was assessed using a checklist consisting of 10 questions with a range of completely undesirable, moderate, and favorable with a score of zero, one, and two, respectively. The total score was calculated between zero and two.

The behaviors were measured by a questionnaire [13]. In this study, FBS was measured by glucose oxidase method by enzymatic colorimetric using glucose oxidase kit (Pars Azmoun company kits approved by Iran’s Ministry of Health and Medical Education). FBS is the most common test used to diagnose diabetes. The test is done in the morning, before the person has eaten. The range of normal blood glucose is between 70 and 100 mg/dl. Levels between 100 and 126 mg/dl are considered as impaired fasting glucose or pre-diabetes [6, 9].

### Intervention

The intervention was designed using TPB to increase healthy lifestyle behavior according to the results of pre-test and cross-sectional study [13]. In this study, the intervention group was divided into 10 subgroups and each group participated in three training sessions and received a TPB-based training program. At the end of the meeting, they were given a booklet. The first session was held for 90 min with the aim of increasing people's awareness and changing misconceptions and strengthening people's true beliefs in the field of healthy lifestyle. Definitions of diabetes and pre-diabetes and ways to prevent diabetes and control blood sugar were given using the lecture. Participants discussed the importance or necessity of physical activity, a healthy diet, and non-smoking.

The second session focused on subjective norms, perceived behavioral control, and behavioral intent for 70 min. Using group discussion, the obstacles and problems of individuals to perform healthy behavior were expressed, then by brainstorming ideas, ways to deal with and control behavior were shared between individuals and used each other’s experience. A list of physical activities that could easily be done daily was also provided, and in order to change the subjective norms, individuals were asked to share the booklet with other family members.

The third session was for 60 min with the overall goal of improving a healthy lifestyle and emphasizing the conclusions, the acceptable and appropriate amount of healthy behaviors. Also, in order to increase people's control over their food intake, a form was provided to them to record all the food consumed per day along with the amount consumed during three different and specified days in a week. In part of the booklet, in order to stimulate increased physical activity, we have dedicated a table for monthly weight and waist recording in which people write down their changes.

In this study, the intervention group was influenced by an educational program designed based on TPB, and the control group received routine health centers training and finally, three months after, using the questionnaire and FBS test the data of both intervention and control groups were collected again and compared with each other.

In this study, the control group received only routine care which included a 12 monthly visit by a doctor in health centers, and public health educators for less than 15 min at a health centers.

### Statistical analysis

Data were analyzed using SPSS software version 26. The distribution of most data was obtained using the abnormal Kolmogorov–Smirnov test. Wilcoxon and paired t-tests were used to compare the mean scores before and after the test to assess TPB variables and healthy lifestyle behavior, Mann–Whitney and independent t-tests and covariance to compare scores between control and intervention groups. Significance level was considered P < 0.05.

### Ethical considerations

After obtaining permission from the Research Council and the University Ethics Committee (IR.ARAKMU.REC.1398.073), we conducted the study in coordination with the city health center. The master's thesis in health education is approved by Arak University of Medical Sciences, Iran and is registered in the Iranian Registry of Clinical Trial (IRCT20190304042921N1).Prospectively registered 22/07/2019, https://en.irct.ir/trial/40596. Before completing the questionnaire, participants were informed about the objectives of the study, they were assured of the confidentiality of information, and then written informed consent was obtained from the individuals. The training package was also provided to the control group after the post-test.

In this clinical trial all the experiment protocol for involving human were in accordance to guidelines of institutional Ethics Committee of Arak University of Medical Sciences, Iran.

## Results

Totally 71 patients (intervention: 36, control group: 35) remained in the study. Findings show that there was not statically significant difference between two groups in terms of demographic characteristics before the intervention (Table 1).

**Table 1:** Comparison of the intervention and control groups, concerning the demographic variables

Group variable	Control (n = 35)		Intervention (n = 36)		*P*-value
	Mean	SD	Mean	SD	
Age (years)	47.91	7.56	46.85	7.42	0.557
Weight (Kg)	76.76	9.34	74.94	12.52	0.490
Height (Cm)	158.19	4.38	158.64	5.95	0.721
BMI (Weight/height (m2))	30.67	3.56	29.58	4.72	0.116

	Frequency (N)	Percent (%)	Frequency (N)	Percent (%)	
*Marital status*					0.149
Married	33	5.7	36	100	
Single	2	94.3	0	0	
*Years of education*					0.448
> 5 years	16	45.7	12	33.3	
5–12 years	16	45.7	21	58.4	
< 12 years	3	8.6	2	5.6	
*Smoking*					0.324
Yes	0	0	1	2.8	
No	30	100	35	97.2	

The mean age of pre-diabetic women in the intervention and control group was 46.85 ± 7.42 and 47.91 ± 7.56 years respectively, which did not have a significant difference based on the results of independent t-test (*p* = 0.557). Other demographic characteristics (qualitative and quantitative variables) of the pre-diabetic women studied are reported in Table 1.

In the control group, the most disorder were high blood lipids (20%) and high blood pressure (Systolic blood pressure higher than 140 mmHg and Diastolic blood pressure higher than 90 mmHg) (17.1%), respectively, and in the intervention group, the most common disorder were thyroid problems (13.9%) and high blood pressure (11.1%), respectively.

The mean scores of awareness and diet-related function in the intervention group increased significantly compared to the control group and the mean FBS in the intervention group significantly decreased compared to the control group (Table 2).

**Table 2:** Comparison of the intervention and control groups, concerning the knowledge and FBS, before and after the intervention

Variables and time	Intervention (n = 36)	Control (n = 35)	P*
*Knowledge*			
Before	0.59 ± 0.17	0.56 ± 0.16	0.247
After	0.73 ± 0.13	0.57 ± 0.17	0.000
P**	0.000	0.632	
*FBS*			
Before	106.56 ± 6.09	107.09 ± 5.36	0.461
After	99.70 ± 11.06	110.94 ± 17.09	0.003
*P*	0.002	0.132	

*Independent T-test

**Paired T-test

Based on the results of Table 2, the mean FBS of the women in the intervention group decreased from 106.56 ± 6.09 to 99.70 ± 11.06 after the intervention (*P* = 0.002), while this decrease was not observed in the control group (*p* = 0.132).

After the intervention, a significant increase was observed in the mean scores of attitude related to physical activity, perceived behavioral control related to physical activity in the intervention group (Tables 3 and 4).

**Table 3:** Comparison of the intervention and control groups, concerning the TPB and diet, before and after the intervention

Variables and time	Intervention (n = 36)	Control (n = 35)	P*
*Attitude*			
Before	4.53 ± 0.42	4.30 ± 0.42	0.023
After	4.64 ± 0.34	4.49 ± 0.46	0.230
Difference between before and after the intervention***	0.10 ± 0.42	0.20 ± 0.49	0.369
*Subjective norms*			
Before	4.52 ± 0.50	4.44 ± 0.52	0.6
After	4.47 ± 0.69	4.30 ± 0.68	0.226
P**	0.609	0.245	
*Perceived behavioral control*			
Before	3.95 ± 0.75	3.75 ± 0.77	0.295
After	4.07 ± 0.69	4.01 ± 0.95	0.912
P**	0.288	0.055	
*Intention*			
Before	4.52 ± 0.77	4.37 ± 0.77	0.304
After	4.61 ± 0.68	4.65 ± 0.53	0.994
P**	0.614	0.090	
*Behavior*			
Before	1.40 ± 0.24	1.25 ± 0.29	0.027
After	1.53 ± 0.20	1.34 ± 0.27	0.003
Difference between before and after the intervention***	0.13 ± 0.27	0.09 ± 0.26	0.456

**Paired T-test

*Independent T-test

***There were the significant differences between the scores mean of attitude and behavior in before between the control and intervention groups, therefore, for the attitude and behavior, the differences between the means of before and after the intervention were compared. Finally, no significant differences were found

**Table 4:** Comparison of the intervention and control groups, concerning the TPB and physical activity, before and after the intervention

Variables and time	Intervention (n = 36)	Control (n = 35)	P*
*Attitude*			
Before	4.42 ± 0.64	4.42 ± 0.38	0.357
After	4.63 ± 0.39	4.53 ± 0.38	0.210
P**	0.047	0.157	
*Subjective norms*			
Before	4.43 ± 0.68	4.28 ± 0.71	0.298
After	4.43 ± 0.71	4.19 ± 0.70	0.089
P**	0.958	0.435	
*Perceived behavioral control*			
Before	3.31 ± 0.86	3.21 ± 0.76	0.606
After	3.34 ± 0.69	3.26 ± 0.93	0.057
P**	0.046	0.770	
*Intention*			
Before	4.22 ± 1.24	4.00 ± 1.23	0.232
After	4.76 ± 0.60	4.45 ± 1.01	0.109
P**	0.056	0.22	
*Behavior*			
Before	0.65 ± 0.83	0.48 ± 0.81	0.3
After	0.48 ± 0.74	0.31 ± 0.63	0.286
P**	0.204	0.201	

**Paired T-test

*Independent T-test

## Discussion

The present study showed that the health education program based on the theory of planned behavior is effective in encouraging pre-diabetic women to promote a healthy lifestyle and improve their fasting blood sugar.

In this study drop outs of samples during study because in the process of creating health behaviors in order to change women attitudes and create stability and sustainability in health behaviors (diet and physical activity), intervention with adequate time in educational sessions are needed therefore drop outs of samples during study. However, to improve some educational content can be delivered indirectly by means of educational booklets, pamphlets, or via social media to reduce the number of training sessions.

In this study, results of the baseline assessment showed that the pre-diabetic women information about healthy life style was 0.059 of 1 score. After three sessions of face-to-face training, the knowledge of pre-diabetic women in the experimental group increased significantly (0.73 of 1 score) in comparison with before the intervention and the control group, which is consistent with the results of the study of Blanks [17]. During the sessions, people were informed about behaviors that reduce the risk of diabetes, and given the role of women in preparing and cooking food, improving knowledge can significantly affect the quality of their family nutrition. After the educational intervention, the attitude related to physical activity in the intervention group increased significantly, which was consistent with the results of the study of Sanaeinasab [18], White [19] who had studied physical activity using the theory of planned-behavior. However, in the present study, in line with White's study [19], no significant increase was found in the attitude of the intervention group regarding a healthy diet. However, the control group's attitude in this regard increased significantly. Completing a questionnaire in this group can affect their belief in the importance of eating healthy food and improve their attitude.

After the intervention, no significant change was found in the subjective norm of the experimental group in relation to physical activity and healthy diet, which contradicts the Maleki's study [20]. Also in Taghipour's study [21], holding training sessions on physical activity for the family and friends of the volunteers, led to the promotion of subjective norms related to physical activity in the intervention group. In the present study, the intervention group was asked to provide an educational booklet to their family members, which we assumed would affect their subjective norms. Therefore, it can be concluded that this method of intervention is unsuccessful in improving the subjective norms of the participants and other methods should be used to improve the subjective norms.

After the educational intervention, the mean score of perceived behavioral control in the experimental group increased, which indicates an increase in the ability of individuals to withstand barriers to physical activity and is consistent with the findings of Sanaeinasab [18].

The literature on people with pre-diabetes shows those participating in self-management and self-efficacy training progress along the continuum necessary for making and sustaining behavior change [19–21].

In the present study, presenting an educational program on factors facilitating the behaviors, providing incentives, reducing and eliminating the perceived barriers, and using the experiences of participant increased the women perceived behavioral control in the intervention group. However, no significant change was found in the perceived behavior control about healthy life style diet in the intervention group. Therefore, it can be concluded that improving healthy diet need to longer an educational session or other methods should be used to improve the perceived behavior control about healthy diet.

Findings show that there was not statically significant difference between before and after intervention in terms of diet in the intervention group because almost TPB construct (attitude, perceived behavior control, intention and knowledge) low increased after intervention.

In the educational intervention, in addition to expressing the benefits of exercise and the harms of inactivity, strategies for adopting sports behaviors and a list of physical activities that can easily be done daily were presented and participants were encouraged to select activities that they could do regularly. However, no significant change was found in the behavioral intention of the intervention group and the average physical activity performance of both groups decreased (although it was not statistically significant), which was consistent with the result of the study of Williams et al. [22] on Physical activity of outpatients using TPB. While Sanaeinasab [18] showed that TPB is effective in promoting physical activity, the reason for this discrepancy could be the cold weather conditions and the global outbreak of coronavirus during the follow-up period in the present study.

Consistent with the study by Chen et al. [23] that a 16-week empowerment program in three phases including awareness, behavior development, and ABC outcomes for pre-diabetic patients significantly improved healthy lifestyles and self-efficacy, in the present study the mean dietary performance in pre-diabetic women in the intervention group was also significantly higher than the control group. After the educational intervention in the experimental group, the mean fasting blood sugar improved in comparison with before the intervention and the control group, which is in line with the study of Shamizadeh [14] using social cognitive theory, Chen’s [23] using ABC empowerment program and Ibrahim’s [24] using a community-based healthy lifestyle intervention program.

The improvement in fasting blood sugar of the experimental group in our study could be due to the improvement in dietary performance in the experimental group. After the intervention, no new cases of diabetes were observed among pre-diabetic patients in the intervention group.

One of the strengths of the present study is investigating the unknown group of pre-diabetics who are difficult to access and at high risk for diabetes. Moreover these finding results can be used by others in the world especially in developing countries (with similar socio-economic status) because theory-based training in the present study can be promotion knowledge and healthy lifestyle in pre-diabetics women.

Among the limitations of the present study are the collection of information by self-report, follow-up of samples, coincided with the prevalence and critical conditions of the coronavirus, and also the lack of predisposing cases in the electronic health record, has made it difficult to identify these people.

It is suggested that future studies should be conducted with other theories and questionnaires with more specific questions about healthy lifestyle behavior and asses the glycemic control with glycosylated hemoglobin test.

## Conclusion

Considering the improvement of knowledge and function of diet and fasting blood sugar after the intervention, theory-based training seems to have beneficial effects. However, other factors such as increasing motivation and removing barriers to promote healthy behavior can be effective. It is also very important to identify and follow up with pre-diabetic people, especially women, to prevent diabetes and its complications, and it is recommended that the health sector focus more on educating these people so that the heavy costs of the disease can be decreased.

## Data Availability

The datasets generated during and analyzed during the current study are available from the corresponding author.

## Abbreviations

TPB: Theory of planned behavior
PAR-Q: The Physical Activity Readiness Questionnaire
FBS: Fasting blood sugar
CVI: Content Validity Index
CVR: Content validity ratio
